# COVID-19 Infection and Previous BCG Vaccination Coverage in the Ecuadorian Population

**DOI:** 10.3390/vaccines9020091

**Published:** 2021-01-27

**Authors:** Daniel Garzon-Chavez, Jackson Rivas-Condo, Adriana Echeverria, Jhoanna Mozo, Emmanuelle Quentin, Jorge Reyes, Enrique Teran

**Affiliations:** 1 Colegio de Ciencias de la Salud, Universidad San Francisco de Quito, Quito CP 170901, Ecuador; dgarzonc@usfq.edu.ec; 2 Ministerio de Salud Publica del Ecuador, Quito CP 170146, Ecuador; jackson.rivas@msp.gob.ec (J.R.-C.); pauliem72@gmail.com (A.E.); johana.mozo@msp.gob.ec (J.M.); 3 Centro de Investigación en Salud Pública y Epidemiología Clínica, Universidad Tecnológica Equinoccial, Quito CP 170527, Ecuador; emmanuelle.quentin@gmail.com; 4 Servicio de Laboratorio, Hospital IESS Quito Sur, Quito CP 170111, Ecuador; jorgereyes83@gmail.com; 5 Facultad de Ciencias Químicas, Universidad Central del Ecuador, Quito CP 170403, Ecuador

**Keywords:** COVID-19, BCG vaccine, BCG vaccine coverage, Ecuador

## Abstract

The Bacillus Calmette–Guérin (BCG) is a well-known vaccine with almost a century of use, with the apparent capability to improve cytokine production and epigenetics changes that could develop a better response to pathogens. It has been postulated that BCG protection against SARS-CoV-2 has a potential role in the pandemic, through the presence of homologous amino acid sequences. To identify a possible link between BCG vaccination coverage and COVID-19 cases, we used official epidemic data and Ecuadorian Ministry of Health and Pan American Health Organization vaccination information. BCG information before 1979 was available only at a national level. Therefore, projections based on the last 20 years were performed, to compare by specific geographic units. We used a Mann–Kendall test to identify BCG coverage variations, and mapping was conducted with a free geographic information system (QGIS). Nine provinces where BCG vaccine coverage was lower than 74.25% show a significant statistical association (χ^2^ Pearson’s = 4.800, df = 1, *p* = 0.028), with a higher prevalence of cases for people aged 50 to 64 years than in younger people aged 20 to 49 years. Despite the availability of BCG vaccination data and the mathematical models needed to compare these data with COVID-19 cases, our results show that, in geographic areas where BCG coverage was low, 50% presented a high prevalence of COVID-19 cases that were young; thus, low-coverage years were more affected.

## 1. Introduction

SARS-CoV-2 was accidentally discovered in China towards the end of 2019, when a cluster of unidentified viral pneumonia cases occurred in Hubei province. During the initial outbreak, the virus was discovered to cause an acute respiratory syndrome, named coronavirus disease 2019 (COVID-19), mostly affecting the elderly (i.e., over 60 years of age) and individuals with underlying health conditions [1,2]. However, the viral mode of transmission and globalization allowed this regional outbreak to spread throughout the world. By spring 2020, SARS-CoV-2 was present in every continent, excluding Antarctica [3]. At the time of writing, SARS-CoV-2 has infected more than 70 million individuals worldwide and has caused nearly 1.2 million deaths [4]. From viral transmission mechanisms [5] to COVID-19 treatments [6], researchers are attempting to understand the disease dynamics better and reduce its burden on humankind. The global spread of COVID-19 cases drives research to attempt to understand how environmental factors may contribute to SARS-CoV-2 transmission and COVID-19 disease severity [7,8]

Along with the spread of the pandemic, most countries imposed a policy of social distancing and other regulations to mitigate COVID-19 [9,10]. However, Ecuador has been the most affected country in South America, with over 200,000 cases and 35,000 excess deaths since February [11]. Among several hypotheses, an association between COVID–19 and the Bacillus Calmette–Guérin vaccine (BCG) has been suggested [12,13]. BCG contains a live attenuated strain of *Mycobacterium bovis*, is widely used to eradicate tuberculosis (TB), and was among the most broadly used vaccinations in the neonatal and young in the twentieth century [14]. Furthermore, BCG is linked with protection against other pathogens, including bacterial infections caused by an antimicrobial response, through the use of toll-like receptors that are able to enhance the expression of Vitamin D receptors on pulmonary epithelial and other cells of the immune system. When Vitamin D receptors join up with calcitriol-activated transcription of antimicrobial peptides [15], in the case of a virus such as yellow fever, BCG creates enhanced activity among Th1 cells [16]. Other mechanisms related to cytokines have been described.

A recent study suggested that enduring innate immune memory is conferred by myeloid cells (monocytes, macrophages, and neutrophils) in response to microbial molecules, metabolic products, or cytokines. Macrophages, which increase effector function (“activation”), are primed for short-term responses (“priming”) or become unresponsive (“tolerance”) [17]. Microbial components can also cause long-term imprinting (“training”) of innate immunity and myeloid cell function, although this is distinct from genomic imprinting, whereby methyl groups are added to DNA in or near-specific genes [18].

Additionally, bioinformatic analysis of BCG antigens showed that bacterial proteins Rv0934, Rv3763, Rv3875, and Rv2997 share common informational properties with the spike protein of SARS-CoV-2, suggesting immunological cross-reactivity which could induce a specific adaptive immune response against SARS-CoV-2. If experimentally confirmed, this theoretical finding could serve as a basis for the design of a BCG vaccine with improved protection against COVID-19 [19].

Moreover, the BCG vaccine has shown a complete response in urothelial bladder cancer in up to 70% of cases. The recommendation of the European Association of Urology and the American Urological Association is to apply the BCG vaccine to patients with intermediate-to-high risk after transurethral resection [20]. Its effects in this therapy are mediated by a fibronectin attachment protein that allows BCG to attach to the disrupted urothelium and become internalized, leading to the activation of urothelium cells and antigen-presenting cells that trigger the production of chemokines and cytokines (especially tumor necrosis factor, IL-6, IL-8, and granulocyte-macrophage colony stimulating factor (GM-CSF)), attracting mononuclear and granulocyte cells to the bladder [21].

Currently, the BCG vaccine is provided to the entire population, in most countries with a high TB incidence [22]. At present, most of the information regarding COVID-19 and BCG come from European countries, the USA, and Australia. Appropriate epidemiological pandemic control and differences in policies regarding the age of vaccination and when countries adopted nationwide vaccination (and whether it was adopted at all) make it impossible to identify a clear correlation. A recent study by Wassenaar et al. indicates the presence of a high attack rate even in countries with high BCG coverage [23]; however, we think that it is extremely important to analyze this phenomenon in more depth, particularly by country or in even smaller geographic units, in order to identify possible correlations and minimize confusion.

In Ecuador, BCG vaccination is still mandatory and free of charge for all newborns [24], but a booster at six years old was stopped in 1999 [25]. As the vast majority of COVID-19 cases in Ecuador were concentrated in adults and young people (20–50 years old) and focused on certain areas of the country, the objective of this report was to correlate the rate of COVID-19 cases adjusted per 10,000 inhabitants with previous exposure to the BCG vaccine 40 years ago.

## 2. Materials and Methods

Ecuador is located in Northwest South America and straddles both the line of the Equator and the Andes Mountains that cross the country from the north to the south and divide it into three continental regions (the Coast, the Highlands, and the Amazon), and there is also one insular region (the Galapagos Archipelago). Geographically, Ecuador comprises 221 cantons that are grouped into 24 provinces and divided into 4 regions, i.e., Coast (7 provinces), Highland (10 provinces), Amazon (6 provinces), and Galapagos Archipelago.

Data on the COVID-19 situation in Ecuador are available online at https://public.tableau.com/profile/direccion.nacional.de.vigilancia.epidemiologica.msp#!/vizhome/COVID19ecu_MSP_DNVE/COVID-19MSP. These data can be disaggregated to the canton level and are updated daily. Data were mapped by using political division by province.

Information on BCG vaccination coverage was obtained primarily from the Ecuadorian Ministry of Health and the Pan American Health Organization. Unfortunately, detailed records at the cantonal level are available only from 2000. Data from between 1990 and 2000 are available only at the province level; until 1979, there were data only at the country level. The BCG vaccination rates 40 years ago (1980), at the province level, were estimated by using a retrospective projection based on the last 20 years with the information available. In addition to the free geographic information system (QGIS) map, a monotonic trend based on the Mann–Kendall statistic was calculated for the BCG coverage, in order to identify changes over time. The Mann–Kendall non-parametric statistical test is used to determine the time trend throughout the annualized vaccination coverage. The test does not require that the data fit any particular distribution. For each spatial unit, the statistic makes combinations of each pair of observed values over time and counts the number of pairs that increase or decrease over time and calculates the relative frequency of increases minus the relative frequency of decreases. Mann–Kendall values range from −1 to +1. When a value approaches +1, it means that there is a monotonic upward trend; when it approaches −1, the trend is downward; and a value of 0 indicates no trend. Statistical analysis: Descriptive analysis, Pearson’s chi-square test, and Student’s *t*-test were performed by using SPSS software version 24, where appropriate.

## 3. Results

**Figure 1:**
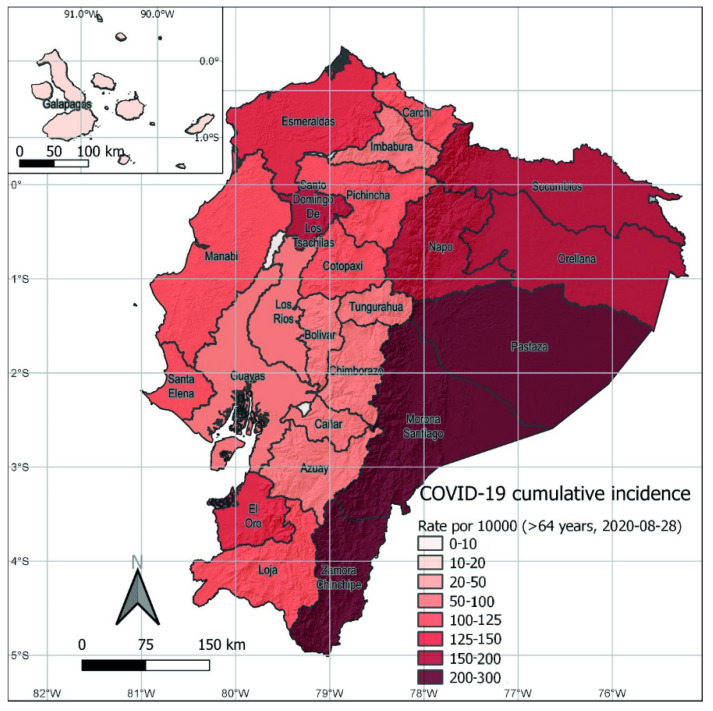
Cumulative incidence of COVID-19 cases adjusted for 100,000 inhabitants, by provinces in Ecuador, by August 2020.

In August 2020, COVID-19 cases were increasing in all provinces. The national case distribution by age groups was as follows: below 20 years (*n* = 5130, 6%), 20 to 49 years (*n* = 59,620, 61%), 50 to 64 years (*n* = 21,167, 19%), and 65 years and older (*n* = 14,155, 13%, Figure 1).

Once prevalence was disaggregated by age, in 17 of 24 provinces (71%), there were more cases per 100,000 inhabitants in people aged 50–64 (Table 1). When provinces were classified by region, the same pattern was found in 70% of the provinces in the Highlands, but was higher in the provinces on the Coast (86%), and slightly lower (67%) in the Amazon provinces (Table 1).

The BCG vaccination coverage in Ecuador that was reported in 1979 was 28%, and it increased in 1980 to 76%. Between 1981 and 1991, BCG coverage ranged from 82% to 99%. After that period, BCG vaccination coverage was stable at around 99% until 2012. Since then, over the last eight years, nationwide coverage has dropped to around 90%.

The analysis from 221 cantons where data were available showed that, in the last seven years, there were 67 cantons (30.6%) with BCG vaccine coverage lower than 50% on average, where nearly one million people live. BCG vaccination coverage between 50% and 89% was found in 90 cantons (41.1%), which have around seven million inhabitants, and only 62 cantons (28.3%) had BCG vaccination coverage over 90%, which included around nine million people, and almost half of these cantons corresponded to urban areas. Additionally, we identified 11 cantons where the BCG coverage was invariably under 50% during the last seven years, with a total of 111,263 inhabitants. The Mann–Kendall analysis showed that BCG vaccine coverage has decreased in 42 cantons during the last seven years, and it has increased in just 14 cantons (Supplementary Materials Figure S1).

Although, as mentioned above, Ecuador currently has 24 provinces, in 1980, there were four fewer provinces: two on the Coast and two in Amazonian regions. In our retrospective projection for the BCG vaccine in 1980, 9 out of 20 provinces had less than 80% coverage. Of those, four were in the Highlands, three on the Coast, one in the Amazon, and one in the Galapagos Archipelago (Table 1).

**Figure 2:**
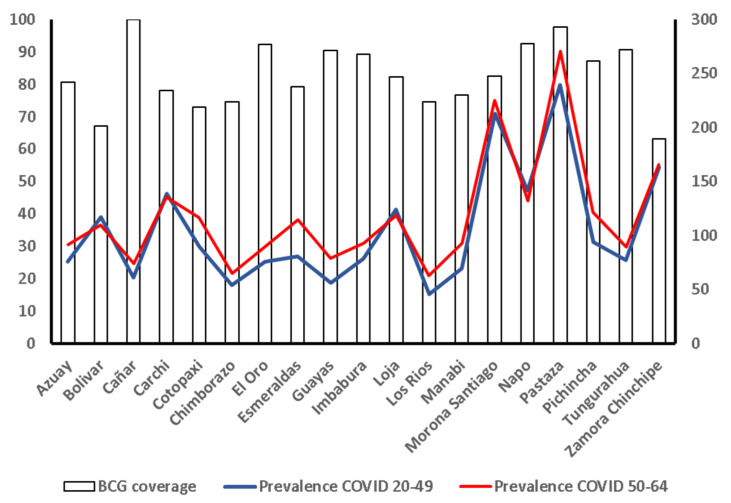
Relationship between BCG vaccine coverage and prevalence of COVID-19 cases, grouped by age, and in all of the 20 provinces in existence in 1980.

**Figure 3:**
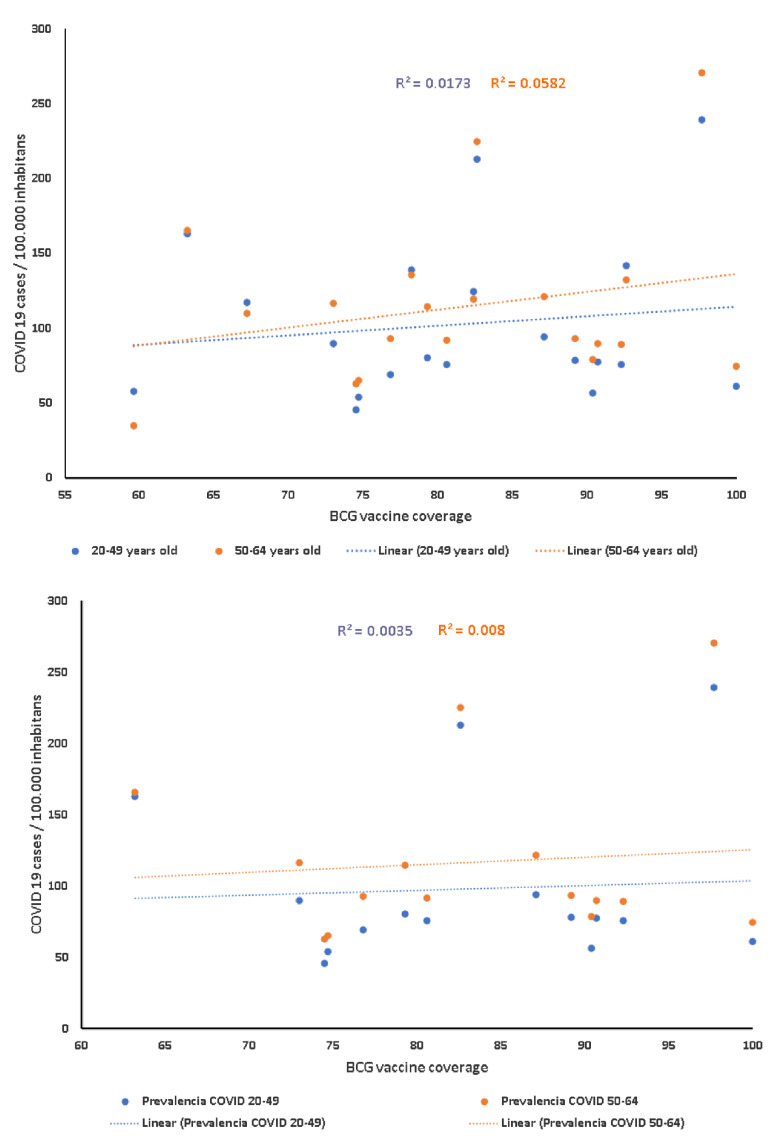
Correlation between BCG vaccine coverage in 1980 (upper panel), provinces with less than 80% of BCG vaccine coverage in 1980 (lower panel), and COVID-19 cases adjusted for 100,000 inhabitants, in August 2020, by age group.

Figure 2 shows the relationship between BCG vaccination coverage in 1980 and the prevalence of COVID-19 cases adjusted for 100,000 inhabitants in patients in the 20–49 and 50–64 age groups. It was found that, although not significant, the correlation between the BCG coverage and the number of COVID-19 cases adjusted for 100,000 inhabitants in the 20 provinces for people aged 50 to 64 years old (r = 0.241, *p*= 0.31) was double that of younger people aged 20 to 49 years old (r = 0.131, *p* = 0.58, Figure 3, upper panel). However, in the nine provinces with a BCG vaccination coverage lower than 80%, the correlation with COVID-19 cases adjusted for 100,000 inhabitants for people aged 50 to 64 years old was r = 0.139 (*p* = 0.78), whereas, in younger people aged 20 to 49 years old, it was r = −0.196 (*p* = 0.53, Figure 3, lower panel).

Comparing provinces with the age distribution of cases (up to the national mean) with BCG retrospective projection coverage below 74.25% shows a significant statistical association (χ^2^ Pearson’s = 4.800, df = 1, *p* = 0.028). However, we did not find a statistically significant association with other age groups or when using the incidence rate. Furthermore, 15 cantons with a low BCG coverage (<50%) presented a high COVID-19 prevalence, over 50 cases per 10,000 inhabitants, with a total population of 6.5 million inhabitants.

## 4. Discussion

It has been proposed that exposure to selected vaccines, such as BCG or microbial components, can increase the baseline tone of innate immunity and trigger pathogen-agnostic antimicrobial resistance, a process known as trained innate immunity, which is directly relevant to resistance against infectious diseases, including COVID-19 [26].

However, as TB prevalence decreased, European countries, including Spain, France, and Germany, stopped their mass vaccination programs, and vaccinating people was only considered necessary for high-risk individuals. Other countries, including China, Russia, and Ukraine, continued national mass BCG vaccination programs. Other countries, such as the United States and Italy, never established national BCG universal programs of vaccination, and only focused on high-risk individuals [11].

In this paper, we explored, from a different perspective, the potential role of childhood BCG vaccination as a determinant factor against SARS-CoV-2 infection in the Ecuadorian population, particularly in those groups with a higher mortality rate, i.e., those aged 50–64. While the members of the 20–49 age group are the most highly exposed, because of their commercial activities, the higher mortality rate in the 50–64 age group could be related to a biological factor, for instance, low BCG vaccination at an early age. Furthermore, this analysis is important because Ecuador was listed as one of the countries where BCG vaccination has no longer been universal or mandatory for more than 10 years, according to the BCG World Atlas [11], in August 2020. This information was unfortunately not accurate. In Ecuador, BCG vaccination for every newborn is a nationwide health policy, and coverage is reported monthly for each of the 221 cantons in the country [12]. Epidemiological information is also provided to the vaccine program of the Pan American Health Organization, and, at the time of writing, the BCG World Atlas was updated to reflect this information [11,13].

The current understanding of broad immune protection mediated by trained immunity and the epidemiologic evidence of long-term protection against viral infections of the respiratory tract, conferred by BCG vaccination, offers a rational biological basis for the potential protective effect of BCG vaccination against severe COVID-19 [14].

In healthy human volunteers, BCG vaccination resulted in enhanced production of pro-inflammatory cytokines (IL-1β, tumor necrosis factor, and IL-6) [15], as well as epigenetic changes resulting in the transcription of genes that are important for antimicrobial responses, and improved cell function, currently known as “trained innate immunity” [16]. This can be seen as a general activity of BCG that could provide protection against SARS-CoV-2; however, a recent study identified a 5 amino acid sequence that is present in virus envelope proteins and displays antigenic properties, as well as the protein Lytr C-terminal, which is highly conserved in Mycobacterium taxa, including *Mycobacterium tuberculosis* and *Mycobacterium bovis* [27]. A recent study by Glisic et al. identified five BCG antigens that correspond to the *Mycobacterium tuberculosis* proteins Rv9034, Rv3763, Rv3875, and Rv2997, which could present a cross-reaction with the S-protein of SARS-CoV 2 [19]. This recent evidence clarified the potential mechanism of how the BCG vaccine could induce specific immunity to SARS-CoV-2.

Previous clinical trials found that BCG revaccination in adults is safe, well-tolerated, and able to boost the immune response of Th1, Th17, and memory NKT-like and NK cells [28,29]. However, the median death rates of COVID-19 for BCG revaccination and other universal immunization country groups were comparable, suggesting that there is no additional benefit of a routine national revaccination policy in terms of decreasing the COVID-19 mortality rate [30]. Currently, BCG revaccination is not recommended by the WHO, and early research has demonstrated no significant evidence in relation to pulmonary tuberculosis, mortality, and extrapulmonary tuberculosis [31].

A recent paper found that the type of BCG policy (i.e., current, interrupted, and never) had a significant effect on COVID-19 deaths per million, with a stronger policy being associated with lower COVD-19 mortality [32]. Additionally, mean BCG coverage (percent) was negatively associated with the COVID-19 deaths per million reported by countries during the first month of the pandemic, where countries with current vaccination programs had lower deaths compared with countries with no or interrupted BCG vaccination programs [33].

Our findings are consistent with an ecological study in 173 countries, including Ecuador, analyzing BCG vaccine coverage (%), using morbidity and mortality as outcomes, which we obtained on an open source basis [34]. That study showed that, among the variety of parameters, “BCG vaccine coverage” had a significant association with COVID-19 mortality, even after adjusting for morbidity, PCR tests, age, universal health coverage, number of medical doctors, elevated total cholesterol, and healthy life expectancy. However, BCG vaccination was not associated with COVID-19 morbidity [34].

On the other hand, another recent study reviewed evidence for a potential biological basis of BCG cross-protection from severe COVID-19, and refined the epidemiological analysis to mitigate effects of potentially confounding factors (e.g., stage of the COVID-19 epidemic, development, rurality, population density, and age structure), demonstrating that every 10% increase in the BCG index was associated with a 10% reduction in COVID-19 mortality [25]. Moreover, a retrospective cohort study, in a limited number of subjects from only one city in the US, suggested that BCG-vaccinated individuals were less likely to require hospital admission during the course of the disease [35]. In contrast to the BCG vaccine, coverage of the measles vaccine, which is also considered to induce heterologous protection against infections through long-term boosting of innate immune responses [36], showed no association with the morbidity and mortality of COVID-19, which was also consistent with a very recent article showing a significantly low risk of COVID-19 mortality in countries with higher BCG vaccine coverage, but not with measles vaccine coverage [12].

These data could suggest a protective effect of BCG vaccination, but such studies cannot provide definitive proof of causality, owing to several inherent biases, including the quality of raw data COVID-19 reports [37], differences in the demographic and genetic structure of the populations, and the non-pharmaceutical interventions being adopted (e.g., quarantine or social distancing, the moment during the pandemic in which the data are analyzed, age of vaccination and changes in this regard (because BCG has better epigenetic activity at an early age, when the body is more susceptible to these changes being made), the time needed for the country to have appropriate coverage, and variations in coverage in terms of geographic areas) [23]. As of 7 November 2020, the effect of prophylactic BCG vaccination on COVID-19 outcomes is being investigated in 18 registered clinical trials (on clinicaltrials.gov). It is important to note that the BCG vaccine leads to lymphadenitis and disseminates disease as a relatively frequent side effect in up to 3% of the population, so whether a large-scale vaccination strategy should be planned in order to achieve a greater advantage (particularly in the presence of this potential risk) should be considered [38].

If the effectiveness of the BCG vaccine against COVID-19 is proven, we are extremely concerned about the impact that this will have in the event of a vaccine shortage, which is what happened at the beginning of the pandemic with other drugs, for instance, hydroxychloroquine for patients with rheumatoid diseases [39]. Currently, there is high global demand for the BCG vaccine. According to the WHO, in 2017, 350 million doses were needed for tuberculosis immunization. It is important to mention that high doses are needed in oncological therapies, and there are a limited number of producers (19 globally); 50% of global demand is covered by only two suppliers, which struggle to keep their prices at a sufficient level to recover production loses. In the case of Ecuador BCG, local production was discontinued for approximately 10 years; therefore, the country depends on the availability of the vaccine in the global market, in order to continue tuberculosis immunizations [40,41].

Unfortunately, the main limitation of this study was the fact that detailed data on BCG vaccination coverage in Ecuador were only available from 1990 and, for the previous decade, the only information accessible was the national consolidates requested by the Pan American Health Organization [12]. As our target population belongs to those receiving vaccination at the beginning of the 1980s, mathematical modeling was necessary to retrospectively project the coverage. Additional risk factors, associated with the elderly, for instance, as well as comorbidities, including hypertension, diabetes, and other chronic diseases, could have been acting as confounding factors, and there is evidence of the association of their presence with the worst evolution of the virus in patients older than 65 who have COVID-19 [42].

Our analysis found that the 15 cantons that currently have a low BCG coverage (<50%) presented a high COVID-19 prevalence, over 50 cases per 10,000 inhabitants, with a total population of 6.5 million inhabitants. However, from our retrospective analysis, it was not possible to demonstrate a relationship between childhood BCG vaccination and actual SARS-CoV-2 prevalence. We were also unable to establish a statistically significant association between cantons where BCG vaccination reductions happened in recent years. Apart from the impact in the form of a reduction in COVID-19 deaths based on BCG vaccination rates from 60 years ago, we believe that our findings in relation to the BCG vaccination coverage reductions in the majority of cantons provide an important index for the Ecuadorian social phenomena during the last decade, such as the important external immigration process and reductions in the size of the health budget [43,44]. Our list of cantons where BCG coverage has suffered from a reduction can be used in the future, to prioritize interventions, to cope with the efforts being made in preparation for the arrival of the COVID-19 vaccine, and it could be used as a potential index of where health-service providers should be reviewed.

Although, before the COVID-19 pandemic, Ecuador allocated 2.78% of its gross domestic product (i.e., USD 3104.8 million) to health coverage [18], it was not able to reach an optimal BCG vaccine coverage in newborn children. This might be because almost 50% of Ecuadorians live in areas where the health sector has been unable to maintain high BCG coverage. We also identified a high prevalence of COVID-19 in these areas [19]. Our findings identified the enormous efforts that will be necessary to improve the Ecuadorian health sector so that it is able to cope with the massive campaign that will be needed once the country has access to the COVID-19 vaccine, including the geographic areas where more work will be required.

## Figures and Tables

**Table 1:** Prevalence of COVID-19 cases (per 100,000 inhabitants) by province and age group, and the calculated coverage of Bacillus Calmette–Guérin (BCG) vaccination in 1980, by province.

Region	Provinces	20–49 Years Old	50–64 Years Old	Coverage
Highlands	Azuay	75.84	91.76	80.6
	Bolivar	117.27	109.73	67.2
	Cañar	61.01	74.38	100.0
	Carchi	139.12	135.45	78.2
	Cotopaxi	89.70	116.58	73.0
	Chimborazo	54.04	65.10	74.7
	Imbabura	78.31	93.17	89.2
	Loja	124.28	119.17	82.4
	Pichincha	94.11	121.36	87.1
	Tungurahua	77.34	89.60	90.7
Coast	El Oro	75.84	89.29	92.3
	Esmeraldas	80.44	114.64	79.3
	Guayas	56.50	78.89	90.4
	Los Rios	45.49	62.99	74.5
	Manabí	69.20	93.09	76.8
	Santa Elena	35.32	77.94	
	Santo Domingo	146.64	145.85	
Amazon	Morona Santiago	213.01	224.96	82.6
	Napo	141.79	132.06	92.6
	Orellana	176.76	153.38	
	Pastaza	239.34	270.66	97.7
	Sucumbíos	131.82	135.10	
	Zamora Chinchipe	163.08	165.61	63.2
	Galapagos	57.53	34.55	59.6

## Data Availability

Publicly available datasets were analyzed in this study. Data about COVID -19 can be found at https://public.tableau.com/profile/direccion.nacional.de.vigilancia.epidemiologica.msp#!/vizhome/COVID19ecu_MSP_DNVE/COVID-19MSP (accessed on 18 December 2020); while BCG vaccination coverage was obtained primarily from the Ecuadorian Ministry of Health and the Pan American Health Organization and is available on request from the corresponding author.

## Supplementary Materials

The following are available online at https://www.mdpi.com/2076-393X/9/2/91/s1. Figure S1: Bacillus Calmette–Guérin (BCG) vaccination coverage in Ecuador, by canton, from 2013 to 2019.
