# Supplementary figures and images for: COVID-19 Infection and Previous BCG Vaccination Coverage in the Ecuadorian Population
Source: Vaccines (Basel). 2021 Jan 27;9(2):91. doi: 10.3390/vaccines9020091 (PMC7912416; doi:10.3390/vaccines9020091)

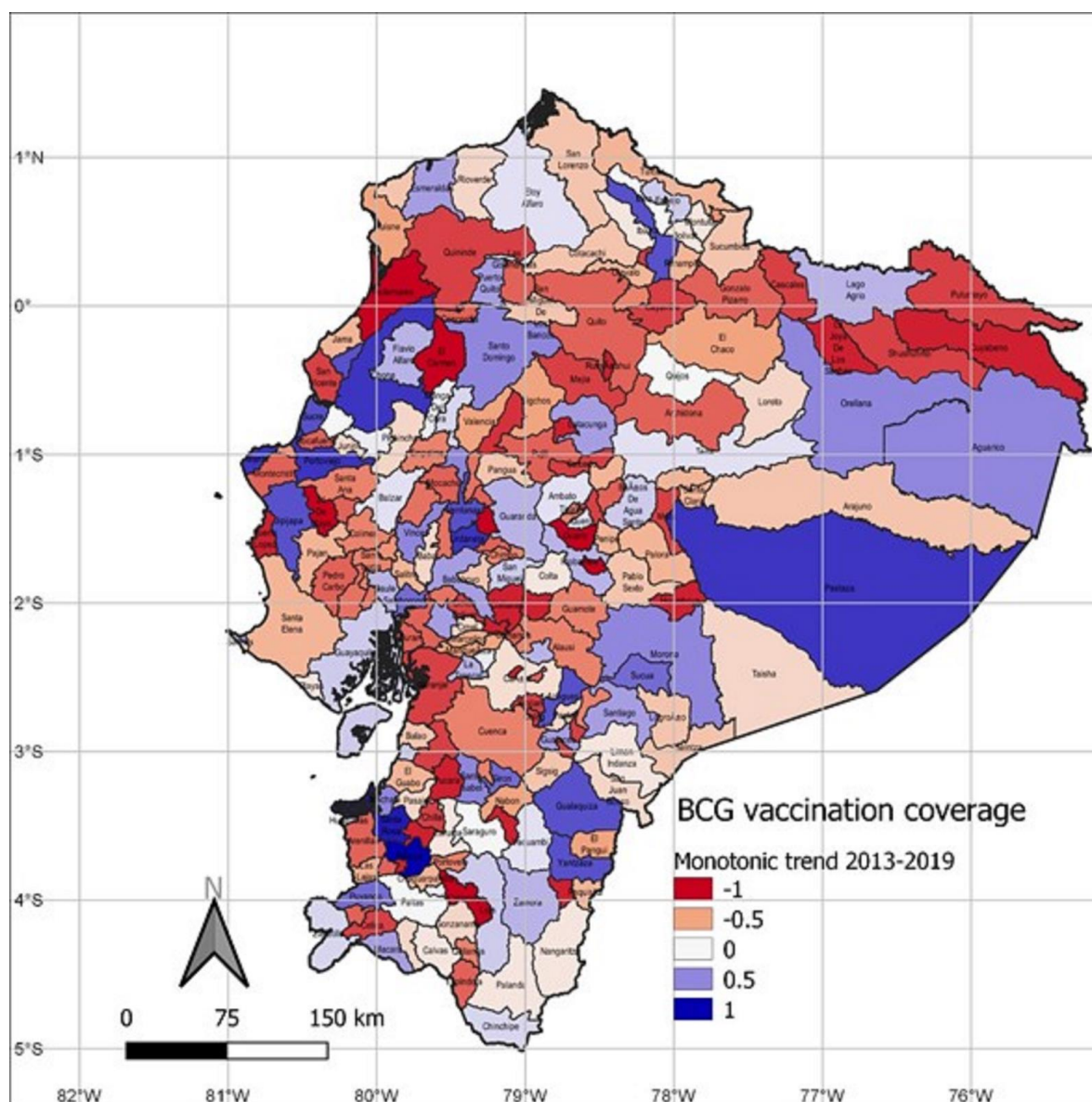

**Figure S1.** Bacillus Calmette–Guérin (BCG) vaccination coverage in Ecuador, by canton, from 2013 to 2019.

Supplement: Supplementary file 1 [file vaccines-09-00091-s001.pdf]
